# The stability of mutualism

**DOI:** 10.1038/s41467-020-16474-4

**Published:** 2020-05-27

**Authors:** Lewi Stone

**Affiliations:** 1 Department of Mathematics, School of Science, RMIT University, Melbourne, Australia; 2 BioMathematics Unit, School of Zoology, Faculty of Life Sciences, Tel Aviv University, Ramat Aviv, Israel

**Keywords:** Ecological modelling, Ecological networks, Population dynamics

## Abstract

Positive interactions are observed at high frequencies in nearly all living systems, ranging from human and animal societies down to the scale of microbial organisms. However, historically, detailed ecological studies of mutualism have been relatively unrepresented. Moreover, while ecologists have long portrayed competition as a stabilizing process, mutualism is often deemed destabilizing. Recently, several key modelling studies have applied random matrix methods, and have further corroborated the instability of mutualism. Here, I reassess these findings by factoring in species densities into the “community matrix,” a practice which has almost always been ignored in random matrix analyses. With this modification, mutualistic interactions are found to boost equilibrium population densities and stabilize communities by increasing their resilience. By taking into account transient dynamics after a strong population perturbation, it is found that mutualists have the ability to pull up communities by their bootstraps when species are dangerously depressed in numbers.

## Abstract (web summary)

Mutualism is typically portrayed as a destabilizing process in community ecology. Here, via a random matrix model that considers species density, the author shows that mutualistic interactions can, in fact, enhance population density at equilibrium and increase community resilience to perturbation.

## Introduction

Mutualism, or cooperative interactions between different species, is an important organizing ecological force that is observed in nearly all living systems, ranging from human and animal societies down to the scale of microbial organisms^1–7^. A general assessment suggests that every species on earth is involved in at least one or more mutualistic interactions^8^. As an example, more than 80% of all flowering plants are in a mutualistic relationship with Mycorrhizal fungi, which live on root systems and enhance the availability of soil^8^. Descriptions of mutualism and cooperation can be traced back to the first recorded historical documents^8–10^, but of note in the twentieth century is the pioneering work of a number of truly colourful characters, including the Russian anarchist Prince Peter Kropotkin^5^, pacifist ecologist Walter Allee^1,11^, and the mathematician Vladimir Kostitzin^12^. Apart from their work, however, there was only relatively minor interest in the study of mutualism over most of last century^13^. Obviously, it did not help that the mathematical models of theoretical ecologists invariably associated unstable positive feedback with mutualistic interactions, often describing exponentially growing populations proliferating in “orgies” of “mutual benefaction”^14^, as coined by May^15^. Such negative associations are often brought in to promote the “Great God of Competition” concept^9,16^, in which competition is viewed as the key source of stability in ecological communities. Although over the last two decades there have been attempts to redress the situation (as in the many ecological studies of Judith Bronstein^8,17^), there is still continued and intense debate on the simple basic question as to whether cooperative interactions between species tend to stabilize, or whether they tend to destabilize the systems they form part of.

Originally May^18^ used “random matrix theory” to explore questions pertaining to the complexity-stability debate^19^, but only in the last years have these sophisticated techniques been adapted to study the role of mutualism in communities^20–25^. Recent key studies in the pages of *Nature* and *Science* also concluded that “mutualistic [interactions] … are destabilizing”^25^ and that increasing the proportion of mutualistic interactions “nearly always decreases the overall return rate” and thus decreases the likelihood of stability^20^, as similarly echoed in the literature elsewhere. In this paper we explore the use of random matrix theory for studying these issues. The analysis shows how mutualism allows species to build up large biomasses or population numbers, and therefore endows the community with strong stabilizing properties. This is particularly noticeable in the short term recovery dynamics, after subsets of species are depressed in population numbers from a strong perturbation. It also corroborates and gives deeper insight into a number of otherwise difficult to explain simulation results and empirical findings of extensive mutualistic interactions sporadically reported in the literature. The results presented here differ from the well-known work of Allesina and Tang^25^, and other similar approaches^20,22^, because here population equilibria are accounted for faithfully in the community matrix, while the latter authors questionably avoid this practice. Moreover, consideration of transient dynamics, gives us a useful framework to help understand the resilience mechanism of ecological communities^26,27^.

## Results

### The random matrix model

The present work is built around a variation of Robert May’s^18^ analysis introduced in the 1970’s to study the controversial question: “Will a large complex system be stable?” Instead of May’s linear dynamics, the classical nonlinear Lotka–Volterra equations of multi-species systems are taken advantage of. For a community of *n*-species, the Lotka–Volterra equations posit that the rate of change of the *i*’th species can be modelled by the nonlinear differential equations:

1 $$\frac{{{\mathrm{d}}N_{\mathrm{i}}}}{{{\mathrm{d}}t}} = N_{\mathrm{i}}\left( {r_{\mathrm{i}} + \mathop {\sum}\limits_{j = 1}^n {a_{{\mathrm{ij}}}N_{\mathrm{j}}} } \right)\quad i = 1,2, \ldots ,n.$$

Here *N*_i_ is taken to be the population density of species-*i*. The interaction coefficients are described in the matrix **A** = (*a*_ij_), where the element *a*_ij_ represents the effect species-*j* has on the growth of species-*i*. **A** is treated as a random matrix with interactions *a*_ij_ assigned randomly having zero mean and standard deviation *σ*, unless otherwise stated. A cooperative or mutualistic (+/+) interaction implies both *a*_ij_ > 0 and *a*_ji_ > 0, while a competitive (−/−) interaction is just the opposite. Exploitative interactions are of the form (+/−).

Intraspecific interactions *a*_ii_ are scaled to unity such that *a*_ii_ = −1. Following many other studies^21,28–34^, to help gain analytical insights into the model’s properties, the growth rates are all given the scaling *r*_i_ = +1, an assumption that is also relaxed in what follows (see Supplementary Notes 3). But in the context of mutualistic communities, a positive growth rate reflects a property of facultative mutualism for species that have the capability of surviving on other resources in the absence of their mutualist partners^1,32,33^. The advantages and disadvantages of this approach are discussed in the “Methods” section.

**Fig. 1:**
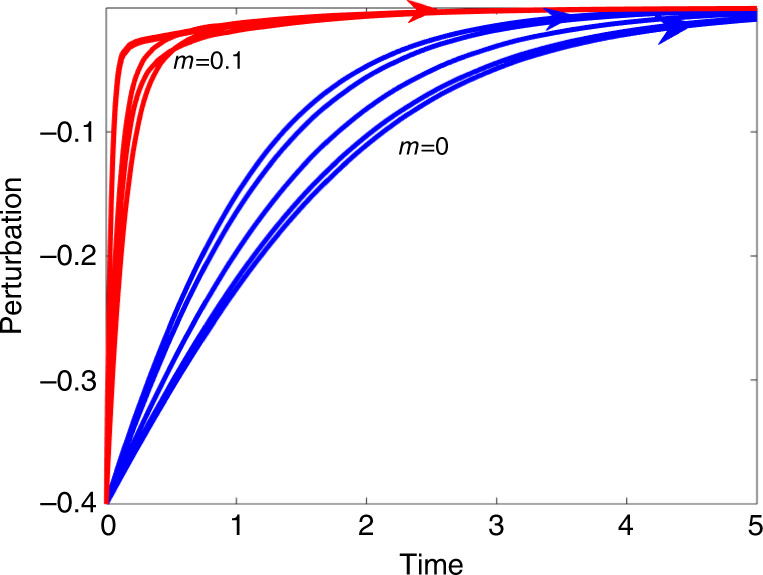
Resilience of Mutualism. Trajectories of a perturbed *n* = 10 species Lotka–Volterra model Eq. (1) as it returns to equilibrium. Blue lines are systems with random positive and negative interaction coefficients having $m = E(a_{{\mathrm{ij}}}) = 0.0\,\,{\mathrm{and}}\,\,\sigma = 0.05$. Red lines are simulations mutualistic systems having random interactions with $m = E(a_{{\mathrm{ij}}}) = 0.1$, $\sigma = 0.05$ and all interactions positive. For all simulations, the population is depressed by 0.4 units initially, and the perturbation dies in time to zero. It is easy to see that the return time is much faster for the mutualistic systems. Note that five independent simulations are plotted for each value of $m$ with identical initial conditions. Supplementary Fig. 1 in Supplementary Notes 2 similarly explores the effects of perturbing some 20 species when there are *n* = 100 species in total.

Before proceeding to the theoretical analysis, it is an appropriate point to introduce directly one of the key phenomena to be investigated. Figure 1 plots trajectories of a perturbed *n* = 10 species Lotka–Volterra model (Eq. (1)) as it returns to equilibrium. The perturbation mimics a species that has been considerably depressed in population numbers over a short time period (sometimes referred to in the ecological literature as a “pulse perturbation”^27,35^). The pulse pushes the system out of equilibrium. One set of simulations (blue lines) has random interaction coefficients with mean strength $m = E(a_{{\mathrm{ij}}}) = 0.0$ and $\sigma = 0.05$ indicating each interaction is equally positive or negative. The second set of simulations (red lines) are of purely mutualistic systems having random interactions with mean $m = E(a_{{\mathrm{ij}}}) = 0.1$ and $\sigma = 0.05$, so that all interspecific interactions are positive ($a_{{\mathrm{ij}}}\, > \, 0$) and mutualistic.

An established and well recognized method for assessing the “degree of stability” of an ecological community requires measuring and comparing the recovery times from such a perturbation^15,27,36^. [The method was employed by May^15^ specifically for mutualist models since at least the 80’s.] From Fig. 1, it is immediately clear that for the perturbed species of the purely mutualistic community (red lines), the return time to equilibrium, is far more rapid indicating far higher resilience. (To assist comparisons, species equilibrium levels have all been translated to appear as zero on the *y*-axis i.e., representing a perturbation of zero.) This much faster recovery time for mutualistic communities stands in contrast to the consensus view which characterizes mutualism as destabilizing, and is counterintuitive to much of what may be encountered in the theoretical ecology literature. But interestingly, a search through the literature reveals that Addicott^32^ had noted the possibility that mutualists have faster recovery time in simulations runs, but this was never explained or explored further by theoretical means. In short then, how can we make sense of this unusual resilience property of mutualistic communities, and is it a characteristic or a pathological occurrence?

### Degree of stability and resilience

Let us analyse model system (1) in further depth. By setting all time derivatives to zero, it is possible to solve for the equilibrium populations $N_{\mathrm{i}} = N_{\mathrm{i}}^ \ast$ The equilibrium is deemed “feasible” if all equilibrium populations are positive $(N_{\mathrm{i}}^ \ast\, > \, 0)$, which is an essential criterion for a viable ecosystem. As noted by Roberts^29^, an unusual feature of this model is that feasible equilibria are nearly always locally stable and will return to equilibrium after a small perturbation, at least for the reasonable scaling of parameters chosen (see also Stone^37^). A full stability analysis requires studying the *n* eigenvalues $\lambda _i$ of the community matrix **S = DA**, where ${\mathbf{D}} = {\mathrm{diag}}(N_1^ \ast ,N_2^ \ast , \ldots .,N_{\mathrm{n}}^ \ast )$ is a diagonal matrix. This is the major difference of the work presented here and the mainstream random matrix methods of Allesina and Tang^25^, who study the eigenvalues of **A** rather than the community matrix **S**.

Following convention, we refer to Λ as the largest eigenvalue of the community matrix **S = DA** i.e., $\Lambda = \max _{\mathrm{i}}\left\{\lambda _{\mathrm{i}}\right\}$. (If the eigenvalues are complex we set $\Lambda = \max _{\mathrm{i}}Re\left\{ {\lambda _{\mathrm{i}}} \right\}$). When Λ *<* 0 the system is locally stable and will return to equilibrium after a small perturbation. However, if Λ > 0, the system is unstable. The degree of the system’s stability or resilience may be quantified by the magnitude |Λ|, which is an index of the system’s return time to equilibrium after a small perturbation^36^. This is based on the knowledge that the eigenvalue associated with Λ is characteristic to the trajectory’s slowest eigendirection on its return to equilibrium. It is therefore considered the bottleneck. The more negative is Λ (the larger is |Λ|), the faster the trajectory can return to equilibrium, and the “more stable” and the more resilient is the system. In summary, when the system is perturbed from a feasible equilibrium by a small perturbation, the “slow dynamics” as the trajectory converges back to equilibrium is controlled by the largest (least negative) eigenvalue Λ and its associated eigenvector.

We will be making use of the Chen and Cohen scheme (see “Methods” section) which generates specially designed interaction matrices **A** in which the proportion of each interaction type is specified in advance. (For example, *P* = 75% mutualists, 15% exploitative and 10% competitive; see also ref. ^20^). Several studies have examined the critical eigenvalue stability index Λ as the proportion *P* of mutualistic interactions is increased. In practice, for each *P*, this requires randomly choosing a proportion *P* of the *n*(*n* − 1) off-diagonal interactions elements $(a_{{\mathrm{ij}}})$ and assigning them to be of type (+/+), while the remainder (1 − *P*) are set to be exploitative (+/−), as explained in the “Methods” section. It was also possible to allow for the network’s connectivity *C* which simply ensures there is a fixed proportion *C* of non-zero interactions.

**Fig. 2:**
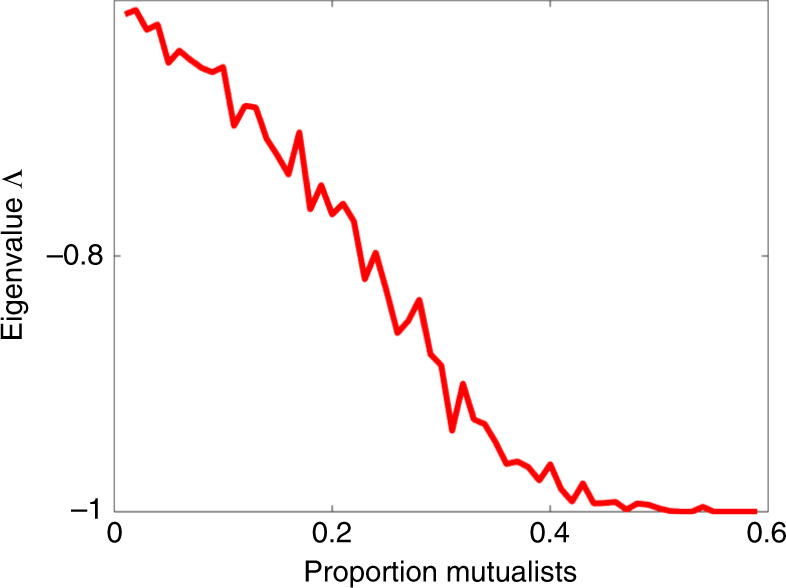
The critical stability eigenvalue $\Lambda$. The eigenvalue Λ is plotted as a function of *P*, the proportion of mutualistic interaction pairs, as an average of 50 runs. The more negative is Λ, the more stable is the (feasible) system in the sense that it has faster return time to equilibrium^36^ and thus higher resilience. Parameters: *n* = 100 species; interaction variability *σ* = 0.02; connectance *C* = 0.7; growth rates $r_{\mathrm{i}} = 1$.

Figure 2 shows that resilience increases, with Λ becoming more negative as the proportion *P* of mutualistic interactions increases, until a saturation point at ${\mathrm{\Lambda }} = - 1$ is reached. In these simulations, all community interactions were taken to be random with an exploitative structure, while mutualistic interactions were externally introduced to the proportion *P* required. But the results were qualitatively unchanged if the background interactions were competitive or purely random.

The relationship is not difficult to explain. As will be shown, in large complex systems as Eq. (1), an increase in the proportion of mutualistic interactions *P* helps build up the equilibrium densities of individual species. For these feasible systems, higher equilibrium numbers translate into stronger stability, similar to a “stability in numbers” effect This link between high equilibrium numbers and strong stability has been demonstrated previously^22,37^. In the context, of feasible competition communities where interactions strengths can reach relatively high levels it has been shown^37^ that $\lambda _{\mathrm{i}} \cong - ( {1 + E( {a_{{\mathrm{ij}}}} )} )N_{\mathrm{i}}^ \ast$. This appears to be different to the result in ref. ^22^ and a derivation is given in Supplementary Notes 1 together with assumptions (e.g., that growth rates $r_{\mathrm{i}}$ = 1, and perturbations of interactions are relatively small as discussed extensively in ref. ^37^).

Feasible mutualist systems have relatively weak interactions, and thus in practice the critical eigenvalue component ${\mathrm{\Lambda }}$ can be well approximated by the minimum equilibrium population $N_{{\mathrm{min}}}^ \ast$, through the relationship:

2 $${\mathrm{\Lambda }} \simeq {\mathrm{max}}\left\{ { - N_{{\mathrm{min}}}^ \ast , - 1} \right\}.$$

(The latter formula takes into account that the stability matrix **S** always has an outlier eigenvalue *λ*_i_ = −1 as explained in the “Methods” section.)

**Fig. 3:**
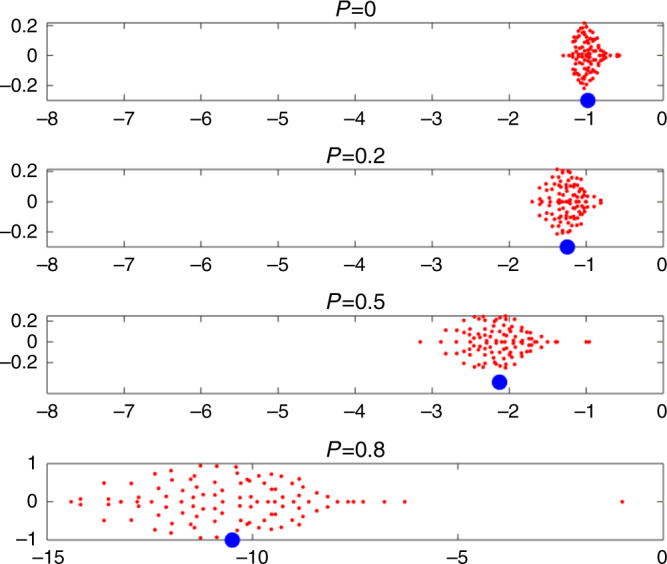
Eigenvalue distributions. Eigenvalues $\lambda _{\mathrm{i}}$ of the Jacobian or community matrix in the complex plane, for four different values of *P*. Imaginary parts Im(*λ*_i_) versus real parts Re(*λ*_i_) are plotted. The “bulk” eigenvalues (red dots) are contained in an ellipse which centers close to the mean equilibrium level, i.e., $- N_{\mathrm{i}}^ \ast$ plotted as blue circles. Stability becomes stronger (ellipse shifts to the left) as the proportion *P* of mutualistic interactions increases in otherwise exploitative communities. When *P* > 0.4, the largest outlier eigenvalue is $\Lambda = - 1$ (see Eq. (2)). For *P* = 0.8, it was necessary to stretch the scale for the *x*-axis and it is different to the other panels. Parameters: *n* = 100 species; interaction variability *σ* = 0.02; connectance *C* = 0.7; growth rates $r_{\mathrm{i}} = 1$, as in Fig. 2.

To explore this further, in Fig. 3 the eigenvalues of the community matrix **S = DA** are plotted for systems of *n* = 100 species and for *P* = 0, 0.2, 0.5, and 0.8. Each subplot gives an elliptical distribution for its 100 eigenvalues $\lambda _{\mathrm{i}}$ in the complex plane (red dots), as predicted by random matrix theory^25^. Adding cooperative interactions (i.e., increasing *P*), pushes the ellipse to the left. The center of the ellipse can be well approximated by the mean equilibrium population level $- N_{\mathrm{i}}^ \ast$ plotted as a blue filled circle, indicating how the equilibrium populations increase in tandem with the eigenvalue distribution (see “Methods” section, Eq. (3)).

A closer examination of the eigenvalue distribution reveals a well known formation in random matrix theory, namely that a multitude of “bulk” eigenvalues are located in the ellipse, while there is an additional “outlier” eigenvalue ${\mathrm{\Lambda }} = - 1$, most easily noticeable for $P \ge 0.4$.

**Fig. 4:**
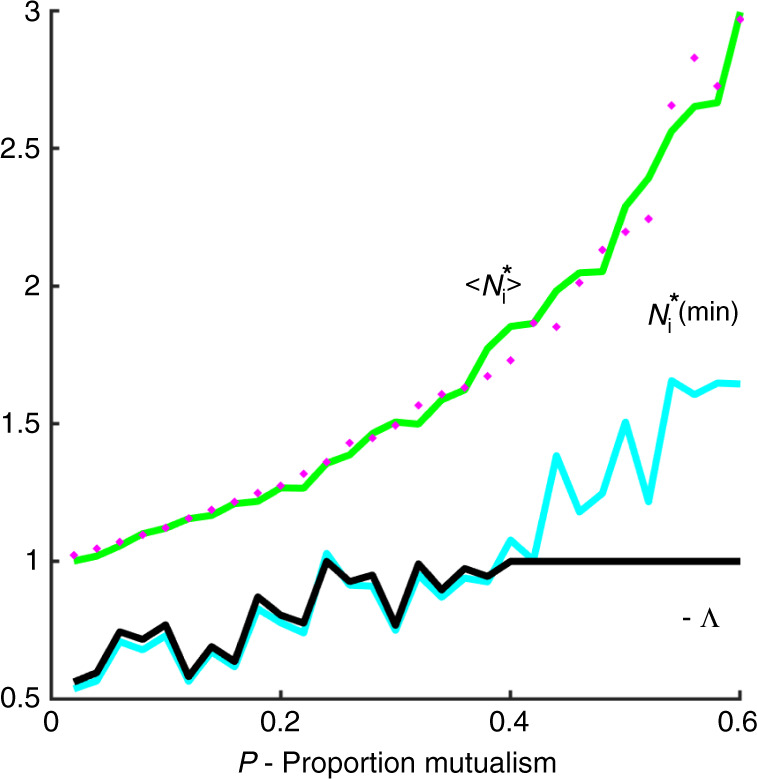
Equilibrium populations. The critical eigenvalue $\Lambda$ (black line) is plotted as a function of *P*, the proportion of mutualists. The minimum equilibrium value $- N_{{\mathrm{min}}}^ \ast$ of the populations (blue line) is a good approximation to the critical eigenvalue $\Lambda$ in the regime $N_{{\mathrm{min}}}^ \ast\, <\, 1$. Outside of this regime $\Lambda = - 1$. Plotted in green is $\langle N_{\mathrm{i}}^ \ast \rangle$ which should be a proxy for the magnitude of the bulk eigenvalues (Eq. (3)). The magenta circles plot the approximation $N_{\mathrm{i}}^ \ast$ ≃ $1/[1 - \left( {n - 1} \right)mCP]$. Parameters, as in Figs. 2 and 3: $n = 100$ species, $\sigma = 0.02$, *C* = 0.7; growth rates $r_{\mathrm{i}} = 1$.

Figure 4 demonstrates that Eq. (2) does indeed hold. For each value of *P*, the equilibrium value of the species with the minimum population is plotted in red. As predicted, the minimum equilibrium value $N_{{\mathrm{min}}}^ \ast$ of the populations (red line) is a good approximation to the critical eigenvalue −Λ (black line) in the regime $N_{{\mathrm{min}}}^ \ast\, <\, 1$ (where *P* < 0.42). Outside of this regime, for higher values of *P*, the critical eigenvalue is then Λ = −1.

### Bulk eigenvalues and short-term recovery

Until now we have examined the system’s return time, and thus resilience, but based on the assumption that the initial perturbation from equilibrium is very small. For larger perturbations, the short term recovery of disturbed populations is controlled by the “bulk eigenvalues” of the stability matrix. To see this, consider Fig. 3 where it is clear that as the proportion *P* of mutualistic interactions increases, the cluster of (*n* − 1) “bulk eigenvalues” associated with the random matrix “moves to the left” and separates out more and more from the “outlier” eigenvalue $\lambda = - 1$. It is interesting to examine how this affects the dynamics of the ecological system. To do so, we will assume that the population of a single species is depressed to a low abundance level by a pulse perturbation^27,35^, as in Fig. 1. The resulting transient dynamics of the population trajectory then roughly passes through two distinct recovery phases.

Phase 1: The rapid first phase response is controlled by the large magnitude “bulk” eigenvalues (rather than the outlier $\lambda = - 1$) which ensure rapid population growth and recovery. These eigenvalues generate the “fast dynamics” and ensure the model’s trajectory rapidly reorients itself towards the direction of the single positive equilibrium vector ${\boldsymbol{N}}^{\mathbf{ \ast }}$ (itself an eigenvector). As this swiftly takes place, the trajectory in any case ends up very close to equilibrium, as seen in Fig. 1. This first phase describes, what was referred to by Arnoldi et al., as the “short term recovery” phase^27^.

Phase 2: Once in the vicinity of the equilibrium, the trajectory is then mostly controlled by the the smallest magnitude outlier eigenvalue, usually $\lambda = - 1$. It then “crawls” at a less rapid pace (along the associated eigenvector) as it converges to the equilibrium ${\boldsymbol{N}} = {\boldsymbol{N}}^{\mathbf{ \ast }}$ at a rate determined by the eigenvalue $\lambda = - 1$, again seen in Fig. 1. This phase describes the longer term recovery time.

Interestingly Phase 2 is traditionally used to measure the return time to equilibrium and the resilience, because, as mentioned, it is perceived as the bottleneck. However, in many cases, as in the context here, it is Phase 1 which is the more relevant measure of recovery and resilience. This is because at the end of Phase 1, the trajectory has almost reached equilibrium, and any remaining discrepancy is in practice negligible.

Thus, with regard to resilience, the critical eigenvalue ${\mathrm{\Lambda }}$ is only the part of the story and represents the speed at which the system returns to equilibrium in the single slow direction of a single key eigenvector. Figure 3 allows us to see that when the proportion *P* of mutualists increases, all the other “bulk” eigenvalues become substantially more negative. The elliptical distribution of the bulk eigenvalues is centered on the average equilibrium population level $- N_{\mathrm{i}}^ \ast$ marked as a blue circle, whose magnitude increases greatly with *P*. Thus the speed to equilibrium from all other (*n* − 1) eigenvector directions increases substantially with higher levels of inter-species cooperation *P*. The short term recovery is thereby controlled by the high magnitude bulk eigenvalues, and is more rapid for larger *P* values.

An easy argument makes it possible to explain why mutualistic interactions tend to increase species equilibrium values $N_{\mathrm{i}}^ \ast$ in this model. As discussed in Stone^37,38^, the underlying “uniform model” (in which all interspecies interaction coefficients $a_{{\mathrm{ij}}} = m$ and $\sigma ^2 = {\mathrm{Var}}(a_{{\mathrm{ij}}}) = 0.0$) acts as a skeleton framework and should be viewed as a good zero’th order approximation when perturbations are small. For the uniform model ($\sigma = 0$), the mean equilibrium value is $N_{\mathrm{i}}^ \ast = 1/[1 - \left( {n - 1} \right)mCP]$, where *P* is the proportion of mutualistic interactions and *C* is the connectance (see Supplementary Notes 1). The RHS of the equation is plotted in Fig. 4 (magenta circles) and provides a close fit to the mean value of the random matrix populations when $\sigma\, > \, 0$. This shows us directly why mutualism (in terms of *P*) builds up the equilibrium populations in the Lotka–Volterra model.

It is also important to note that going beyond a threshold of too many mutualistic interactions can lead to population blowup and loss of feasibility. To see this in simple terms, suppose all interactions are mutualistic of strength $a_{{\mathrm{ij}}} = + m\, > \, 0$. The assumption leads us to the “regular” model in which all species are identical. The model Eq. (1) at equilibrium yield: $N^ \ast = 1/[1 - \left( {n - 1} \right)m]$ for the case where *C* = *P* = 1 (see Supplementary Notes 1). Clearly, we must have the limitation $m\, <\, 1/\left( {n - 1} \right)$ to prevent blow-up, and feasibility is lost if there is equality (see refs. ^37,38^). These are important factors that constrain the feasibility of mutualistic systems.

## Discussion

In summary, for the parameters discussed here, feasible systems based on the Lotka–Volterra Eq. (1) are generally locally stable, and the strength of stability increases with *P* the proportion of mutualists. The unusual results presented are based on studying the eigenvalues of the complete community matrix **S = DA**, rather than just the interaction matrix **A**, as has been recent practice^20,25^. The latter approach fails to consistently take into account species heterogeneous equilibrium levels, which has a number of consequences, including overlooking the startling resilience properties of mutualists systems found here.

Given that the models studied in this paper have the interesting property that $\lambda _{\mathrm{i}} \simeq - N_{\mathrm{i}}^ \ast$, one can expect in practice that rarer species will take more time to recover that abundant species, a feature predicted heuristically in ref. ^27^. While the latter authors “emphasize that there is no mathematically inevitable link between species rarity and long-term return rates,” in contrast we see here that there is a formal mathematical link. Namely, the eigenvalues of the community matrix **S = DA** control the return rates and these are in turn proportional to population abundance $\lambda _{\mathrm{i}} \simeq - N_{\mathrm{i}}^ \ast$. This leads to the interesting observation of Arnoldi et al.^27^ that abundant species tend to govern the short-term recovery, while rare species often dominate the long-term recovery.

The results indicate underlying generic stability properties of mutualism that have been observed in simulation studies in the past, but have until now defied explanation from a theoretical perspective^21,39,40^. Note that there has been no need to introduce nonlinear functional responses to explain the stability of mutualism to capture this generic property. Additional nonlinearities are likely to make these results even more robust, as has been shown elsewhere^21^. That mutualism might in a number of contexts be stabilizing is not out of line with Tu et al.^24^ conclusion that there are increasing and even “widespread” reports of mutualistic interactions (or other cooperative interactions) in many natural and laboratory communities^41,42^ and where biodiversity is very high.

A possible caveat with the present approach is the narrow range of parameters that allow for a feasible mutualistic system. This is seen in Supplementary Notes 5 where general calculations are given for estimating the probability of feasibility, based on refs. ^10,37^, while Supplementary Fig. 5 verifies the accuracy of these calculations. We see that for *n* < 100, the feasibility constraint is not that dissimilar to May’s stability condition for the stability of large random ecosystems (namely, $\frac{{n\,\sigma ^2}}{{(1 + m)^2}}\, <\, 1$), and neither is it dissimilar to the feasibility requirements of competition systems or other related random matrix analyses from the literature. As discussed above, the other requirement that the underlying “uniform model” is feasible sets the limitation on mutualistic interaction strength as $m\, <\, 1/\left( {n - 1} \right)$. In short, large feasible systems require that species interact weakly and with low variability. If these conditions are met, large populations can build up and exhibit very high stability properties. In practice these requirements are likely to be far looser. Sequential assembly of communities should effectively select for feasible communities, and thus mitigate the above-mentioned caveat. Thus even if feasibility is rare amongst all community matrices, it may well be easier to generate in nature. However, this is a small diversion from the guiding questions in this study which concern the features controlling the degree of stability of mutualistic systems.

While the model presented here, like all models, requires some simplifying assumptions, it nevertheless has many commonalities with those used in the past 40 years for studying the complexity-stability debate^18,25,38^. As shown in Supplementary Notes 3, some of the assumptions can be relaxed without damage to any of the results presented. Although not necessarily a universal principle (Supplementary Notes 4), the general examples here demonstrate that cooperative interactions between species have the propensity to boost the stability of feasible systems. In short, mutualists have the ability to pull up communities by their bootstraps, should species become depressed in number or pushed out of equilibrium.

## Methods

### Stability

Local stability of the Lotka–Volterra Eq. (1) at equilibrium is found from studying the community matrix, or equivalently Jacobian, defined as ${\mathbf{S}} = {\mathbf{DA}}$, where ${\mathbf{D}} = {\mathrm{diag}}(N_1^ \ast ,N_2^ \ast , \ldots .,N_{\mathrm{n}}^ \ast )$ is the diagonal matrix of equilibrium populations $N_{\mathrm{i}}^ \ast$. This requires calculating the eigenvalues $\lambda _{\mathrm{i}}$ of **S = DA** . Following convention, we refer to Λ as the largest eigenvalue of the community matrix **S = DA** i.e., $\Lambda = \max _{\mathrm{i}}\lambda _{\mathrm{i}}$. If the eigenvalues are complex we set $\Lambda = \max _{\mathrm{i}}Re\left\{ {\lambda _{\mathrm{i}}} \right\}$. As is well known, the equilibrium of system (1) is locally stable if $\Lambda \, < \, 0$.

### The population eigenvector ${\boldsymbol{N}}^{\mathbf{ \ast }}$

Another useful property of Eq. (1) under the given scaling is the equilibrium condition **S**${\mathbf{N}}^ \ast = - {\mathbf{N}}^ \ast$ (see ref. ^38^). To see this, consider a feasible equilibrium ${\boldsymbol{N}}^{\mathbf{ \ast }}\, > \; 0$. Then according to Eq. (1), at equilibrium ${\mathbf{A}}\,{\boldsymbol{N}}^{\mathbf{ \ast }} = - {\boldsymbol{r}} = - {\boldsymbol{e}}$. Recall that the stability matrix is $\mathbf{S}= {\mathbf{DA}}$. Thus ${\boldsymbol{SN}}^{\mathbf{ \ast }} = {\mathbf{DA}}\,{\boldsymbol{N}}^{\mathbf{ \ast }} = - {\boldsymbol{De}} = - {\boldsymbol{N}}^{\mathbf{ \ast }}$, and the vector ${\boldsymbol{N}}^{\mathbf{ \ast }}$ must be a right eigenvector of **S**. This assumes that the birth rate eigenvector is unity, i.e., ${\boldsymbol{r}} = {\boldsymbol{e}}$.

The above property ensures there is always an eigenvalue $\lambda _{\mathrm{i}} = - 1$ associated with the eigenvector ${\boldsymbol{N}}^{\mathbf{ \ast }}$ (which is positive if the system is feasible), and it is often the largest eigenvalue Λ, as seen in Fig. 2. For purely mutualistic systems (all species interactions are positive), the Perron-Frobenius theorem ensures that $\Lambda = - 1$. [One sees this from studying the matrix **S**=−***αI*** + ***P*** where ***α*** >− min{**S**_ii_}—details given in Supplementary Notes 5].

### Resilience and transient dynamics

Based on the eigenvalues of **S**=**DA**, if $\Lambda = \max _{\mathrm{i}}{\mathrm{Re}}\left\{ {\lambda _{\mathrm{i}}} \right\}\, <\, 0$, then the degree of the system’s stability or resilience is traditionally quantified by the magnitude |Λ|. The latter is an index of the system’s return time to equilibrium, after a small disturbance. Note that Λ is characteristic to the slowest eigendirection of the trajectory as it returns to equilibrium and is often considered the bottleneck. Conversely, the more negative is Λ (the larger is |Λ|), the “more stable” is the equilibrium of Eq. (1), and the more resilient is the system. When the system is perturbed from equilibrium by a minute amount, the “slow dynamics” are controlled by the largest eigenvalue associated with Λ and its eigenvector. However, when perturbations are more substantial, there is an interplay between the fast and slow dynamics associated with Phase 1 and Phase 2, respectively, as described in the main text.

Note also that the trace properties of the community matrix imply $\mathop {\sum }\nolimits_{i = 1}^n - \, N_{\mathrm{i}}^ \ast = \mathop {\sum }\nolimits_{i = 1}^n \lambda _{\mathrm{i}}$, and thus

3 $$\left\langle { - N_{\mathrm{i}}^ \ast } \right\rangle = \left\langle {\lambda _{\mathrm{i}}} \right\rangle .$$

This explains why the elliptical eigenvalue distributions in Fig. 3 appears centered on $\langle - N_{\mathrm{i}}^ \ast \rangle$.

### Growth rate parameters $r_{\mathrm{i}}$

The approach advocated here monitors the model’s stability while allowing the parameter *P*, the proportion of mutualists, to increase. All other parameters are rigidly fixed to constants. Then we can be sure only mutualism *P* is responsible for any changes observed in the model’s dynamics. Had other parameters, for example the growth rates $r_{\mathrm{i}}$ changed as well, it would be extremely difficult to untangle whether it was the rates $r_{\mathrm{i}}$ or whether it was the proportion of mutualists *P*, or both, that were responsible for any change in stability.

This should be contrasted with some other methods^43^ that have instead advocated keeping equilibrium numbers conveniently normalized to unity $(N_{\mathrm{i}}^ \ast = 1)$ and which opens the door for allowing for a wider range of intrinsic growth rates, including $r_{\mathrm{i}}$ both positive and negative in value (rather than fixing them rigidly to $r_{\mathrm{i}} = + 1$). However, the latter method has the disadvantage of changing the growth rates $r_{\mathrm{i}}$ every time *P* is changed. As discussed above, changing other parameters as the proportion of mutualism *P* is increased, makes it difficult to pinpoint exactly what is responsible for changes in stability. (Is it *P* or changes in *r*_i_?)

In contrast, when the growth rates are all fixed to $r_{\mathrm{i}} = + 1$, as implemented in this paper, Figs. 3 and 4 show that the higher is the proportion of mutualism *P*, the higher is the mean equilibrium density $N^ \ast = \left\langle {N_{\mathrm{i}}^ \ast } \right\rangle$ (the blue dots plot $- N^ \ast$). Since the ellipse of the system’s eigenvalues is approximately centered on $- N^ \ast$, adding cooperative interactions pushes the ellipse to the left, increasing stability. This now can only be attributed to the increase in *P*.

Nevertheless, the method of forcing all equilibrium populations to unity $(N_{\mathrm{i}}^ \ast = 1)$ has the advantage of studying a different class of feasible systems for which $N_{\mathrm{i}}^ \ast = 1$ and including both positive and negative birth rates. As many ecosystems have species which critically depend on their interactions with other species for survival, having negative growth rates is a realistic option. But choosing this option in which all $N_{\mathrm{i}}^ \ast = 1$, creates yet other difficulties. In the context of this paper, it implies that all species have the same recovery time characteristics, making it difficult to explore the properties of short and long-term recovery times. Historically, many theoretical ecologists^21,28–34^ have gone with the option of setting $r_{\mathrm{i}} = 1$. But the recent interest in freeing the growth rates and fixing the equilibrium populations presents an interesting alternative picture that needs to be explored further^43^ (see eg., Supplementary Notes 3).

### Interaction parameters

In the simulation experiments shown in Figs. 2–4, we make use of Chen and Cohen’s scheme^43^ for generating interaction matrices **A**. Their scheme makes it possible to specify a predefined proportion *P* of mutualists, by randomly choosing a proportion *P* of the *n(n* − *1)* interactions $(a_{\mathrm{ij}})$ and assigning them to be of type (+/+). Each of the proportion *P* interactions was drawn from a half normal distribution $|N\left( {0,1} \right)|$. The remainder (1 − *P*) “background interactions” were set to be exploitative (+/−) based on the half normal distribution as well. Other interaction pairs types (e.g., competitive) were also examined for background interactions^20^.

### Linearization

The analysis here largely relies on studying a linearization of Eq. (1) about equilibrium, and will only be numerically accurate as long as the linearization gives a reasonable representation of the dynamics. Although this may not be true for very large perturbations, the potential limitation has not yet been observed in numerical simulations. Figures 1 and 4 and Supplementary Figs. 1–4 for example, all show the expected qualitative behavior despite the relatively large perturbations.

### Reporting summary

Further information on research design is available in the Nature Research Reporting Summary linked to this article.

## Supplementary information

Supplementary Information

Reporting Summary

## Data Availability

All data in all four figures was generated from Matlab code available in github repository https://github.com/lewistone/Mutualism.

## Code availability

Numerical computations were performed using Matlab. Complete code for generating all four figures appearing in the article is given in the following publicly available github repository: https://github.com/lewistone/Mutualism.
