# Supplementary material for: The stability of mutualism
Source: Nat Commun. 2020 May 27;11:2648. doi: 10.1038/s41467-020-16474-4 (PMC7253468; doi:10.1038/s41467-020-16474-4)
Supplement: Supplementary file 1 — Supplementary Information [file 41467_2020_16474_MOESM1_ESM.pdf]

The Stability of Mutualism

Stone[1,2], L.

[1]Mathematics, School of Science, RMIT University, Melbourne, Australia;

[2]BioMathematics Unit, Dept. of Zoology, Faculty of Life Sciences, Tel Aviv University, Ramat Aviv, Israel.

Email: lewistone100@gmail.com

Supplementary Notes

Supplementary Notes 1 :   Stability Matrix S

Connection between eigenvalues of S and population equilibrium values N_i^*: Following Stone (2016,2018)[1,2] it is possible to sketch why the eigenvalues of **S** are closely related to the equilibrium populations N_i^* of Equation (1). As before, it is assumed that growth rates r_i=1. By definition

$$S = DA = D[-I + M + B] = D[(-1 - m)I + B + me^T e] .$$

Here **D**=*diag(Ni*)* and **A**=*(aij)* is the matrix of interactions, and all n^2 elements of **M** are $m_{ij}=m$. **B** is the matrix of random perturbations, and $e = [1,1, \dots ,1]'$. Stone (2016,2018) showed that $\mathbf{D}e^T e$ is a rank-one perturbation of **S** with a special property that ensures the eigenvalues of **S** are (apart from the outlier λ_1=-1) exactly equal to the eigenvalues of S':

$$S' = D[(1 + m)I + B] = -(1 + m)D[I + B']$$

where $B' = B/(1 + m)$.

As discussed in Stone, for $\gamma < 1$, based on an off-diagonal perturbation the (n-1) eigenvalues of $\mathbf{S}'$ and thus **S** are

$$\lambda_i(S) \simeq -(1 + m)N_i^* \qquad \text{i=2,3,…,}n,$$

while there is also an outlier eigenvalue: $\lambda_1(S) = -1$.

The approximation is based on the assumption that the mean strength of mutualism m is small and that the perturbations are relatively small, namely $\gamma^2 = \frac{n\,\sigma^2}{(1+m)^2} < 1$, which is generally the case for feasible mutualistic systems. These points are discussed extensively in Ref.[2]

For the Chen & Cohen model with proportion P of mutualists in an exploitative community, the eigenvalues can be approximated as:

$$\lambda_i(S) \simeq -(1 + mCP)N_i^* \qquad \text{i=2,3,…,}n,$$

where for mutualistic interactions $E(a_{ij}) = E(a_{ji}) = m,$ and C is the connectivity while P is the proportion of mutualistic interactions. For feasible systems m is in any case small in practice ($m \ll 1$), and a reasonable approximation may be given by:

$$\lambda_i(S) \simeq -N_i^* \qquad \text{i=2,3,…,}n,$$

while the outlier eigenvalue remains: $\lambda_1(S) = -1$.

Equilibrium of the uniform model. For general calculations note that with proportion P of mutualists in an exploitative community, the mean equilibrium population can be approximated by

$$< N_i^* >= 1/[1 - (n - 1)CPm]$$

where for mutualistic interactions $E(a_{ij}) = E(a_{ji}) = m$. Here C is the connectivity and P the proportion of mutualistic interactions. This is because for the uniform model, every species behaves as every other, so that $N_i^* = N^*$ at equilibrium. Eqn.1 at equilibrium then gives:

$$-N^* + \sum_{i=1}^{k} mN^* = 1$$

where k=$(n - 1)CP$, and thus giving the expression above for $< N_i^* >$.

We can check the accounting based on the trace formula: Trace(**S**)= $\sum_{i=1}^{n} N_i^* = \sum_{i=1}^{n} \lambda_i$.
For the uniform model $N_i^* = 1/[1 - (n - 1)mCP]$, and thus

$$\sum_{i=1}^{n} \lambda_i = -(n-1)(1+mCP)N_i^* - 1 = \; -n/[1-(n-1)mCP] = -\sum_{i=1}^{n} N_i^* \;,$$

which corroborates the trace formula for the uniform model.

Stability and eigenvectors of the matrix S for systems of pure mutualism: For purely mutualistic systems in which all inter-species interactions are positive, the Perron-Frobenius theorem ensures that $\Lambda = -1$. One sees this from studying the matrix $\mathbf{S} = -\boldsymbol{\alpha I} + \boldsymbol{P}$ where $\boldsymbol{\alpha} > -\min\{\mathbf{S}_{ii}\}$ is chosen so that the matrix **P** is nonnegative. Thus **P** satisfies the Perron-Frobenius theorem, with

$$\boldsymbol{P}\mathbf{N}^* = \; \boldsymbol{S}\mathbf{N}^* + \; \boldsymbol{\alpha}\mathbf{N}^* \;\; = (-\mathbf{1} + \boldsymbol{\alpha})\mathbf{N}^*.$$

According to the theorem the spectral radius of **P** is $(-\mathbf{1} + \boldsymbol{\alpha}) > 0$, since **P** also has the *positive* eigenvector $\mathbf{N}^*$ when the system is feasible. For any eigenvector **x**, $\mathbf{S}\boldsymbol{x} = (-\boldsymbol{\alpha}\mathbf{I} + \boldsymbol{P})\;\mathbf{x}$, and given we know the spectral radius of **P,** we see the largest eigenvalue of **S** must be $\Lambda = -1$ and also has the corresponding positive eigenvector $\mathbf{N}^*$ **.**

Supplementary Notes 2 : Perturbing n=100 species

Supplementary Figure 1 complements Figure 1 in the main text but for *n*=100 species of which 20 are perturbed. (Fig.1 has *n*=10 species only.)

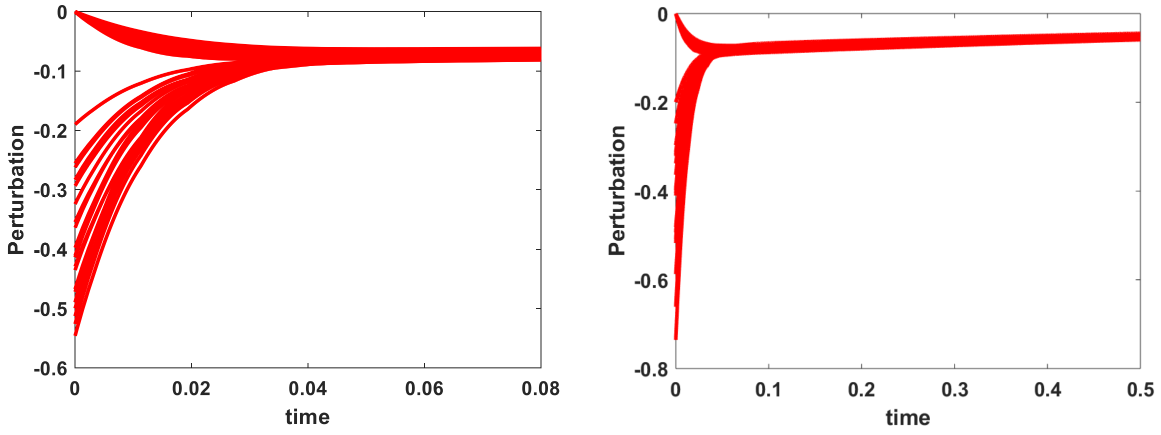

Supplementary Figure 1. Trajectories of a perturbed *n*=100 species Lotka Volterra model (Eqn.1) as it returns to equilibrium. Red lines are simulations mutualistic systems having random interactions with $m = E(a_{ij}) = 0.01$ and $\sigma = 0.005$. Twenty of the populations are depressed by approximately 0.4 units initially, and the perturbation dies in time. LHS shows fast time dynamics up to t=0.08. RHS shows the perturbation slowly decaying to zero

As noted by a reviewer, a pathological counter-example of the model predictions would occur if a (small) proportional perturbation of all species would be along the main eigenvector, thus would mostly show the effect of the lead eigenvalue. In contrast a (small) perturbation of a subset of species should display the effect of the fast eigenvalues first (as in fact shown in Figure 1). _____

Supplementary Notes 3: Simulations with random and negative growth rates

1) In this section of the SI, it is shown that the resilience properties are unchanged when the constancy assumptions on the intrinsic growth rates r_i are relaxed. Thus Supplementary Figure 2 below repeats five simulations as in Figure 1 in the main text except that for this n=10 species community, I have taken ALL growth rates to be $r_i \sim$ Uniform (0,1) [i.e., random draws from a uniform distribution between 0 and 1]. Again, the conclusion is the same as in the main text. The higher the level of mutualism the faster the return time and short term recovery. Five simulations are shown for each m value.

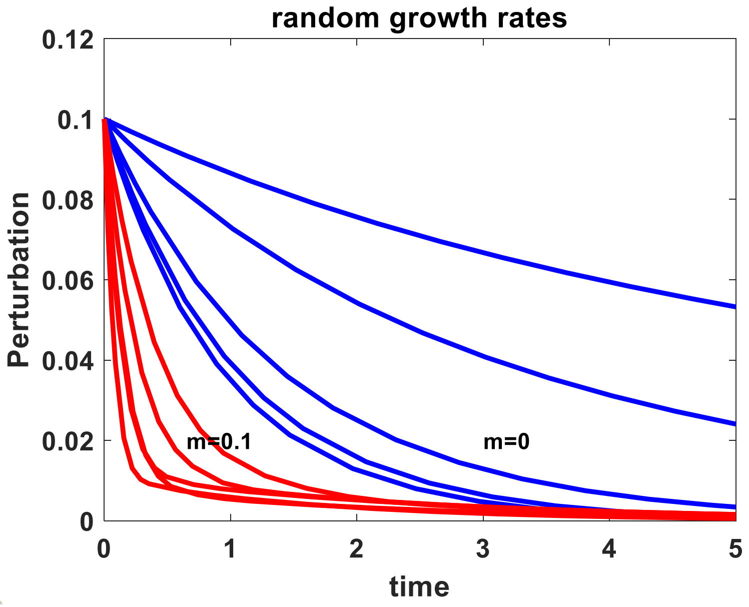

Supplementary Figure 2. Blue lines are systems with random interaction coefficients having $m = E(a_{ij}) = 0.0$ and $\sigma = 0.05$. Red lines are simulations mutualistic systems having random interactions with $m = E(a_{ij}) = 0.1$ and $\sigma = 0.05$. For each value of m there are five simulations as in Figure 1 in the main text except that for this *n*=10 species community, all growth rates are taken to be $r_i \sim$ Uniform (0,1).

2) In Supplementary Figure 3 below, I show results of a *n*=10 species community where one species has a negative growth rate r_1 =-0.05 and all other species have r_i =+1 set at unity. The standard deviation of interactions are σ=0.05. Again, the conclusion is the same as in the main text. The higher the level of mutualism the faster the return time and short term recovery. A single simulation is shown for each *m* value.

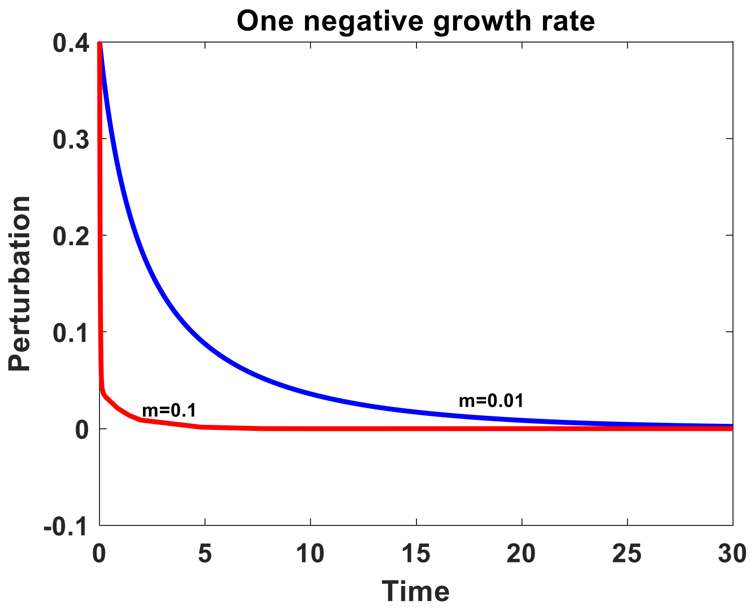

Supplementary Figure 3. Blue line: a simulation with random interaction coefficients having $m = 0.01$ and $\sigma = 0.05$. Red line $m = 0.1$ and $\sigma = 0.05$. For an n=10 species community, one species has a negative growth rate r_1 =-0.05 and all other species have r_i =+1 set at unity.

3) In Supplementary Figure 4 below, shows results for an n=10 species community where two species have negative growth rates r_1 =-0.05 And r_2 =-0.05 and all other species have r_i =+1 set at unity. The standard deviation of interactions is σ=0.05. Again, the conclusion is the same as in the main text. The higher the level of mutualism the faster the return time and short term recovery. A single simulation is shown for each m value.

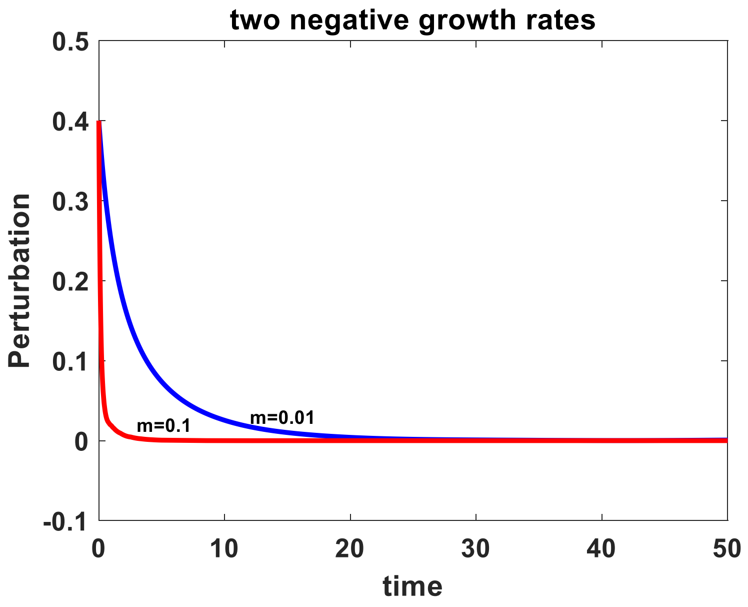

Supplementary Figure 4. Blue line: a simulation with random interaction coefficients having $m = 0.01$ $\sigma = 0.05$. Red line $m = 0.1$ and $\sigma = 0.05$. For an n=10 species community, two species have negative growth rates r_i =-0.05 and all other species have r_i =+1 set at unity.

Supplementary Notes 4: Relation to the paper of Stone (2016)[1]

In Figure 3 of Stone (2016), the equilibrium abundance of some species decrease as mutualism *m* increases. However, in Figure 4 of the present paper, the equilibrium abundance increases as the fraction of mutualism increases. This calls for some explanation.

First, Stone (2016) provides an analysis of a Competition-Mutualist (C-M) model whereas the main model analysed in this paper is one of pure or close to pure mutualism, and thus very different. For the C-M model there are two sets of species, plants and their pollinators. There is competition (strength *c*) between the different plant species and competition (strength *c*) between the different pollinator species. But all plant-pollinator interactions are mutualistic of strength m. The adjacency matrix of the interaction network is bipartite in structure. Altogether, this creates a far more complex situation.

Thus the two models in the two papers are quite different. Below I have reproduced Supplementary Figure S19a&b from Stone (2016). Note that a) also corresponds to Fig.3 in the main text of Stone (2016) where some equilibrium abundances decrease with *m*

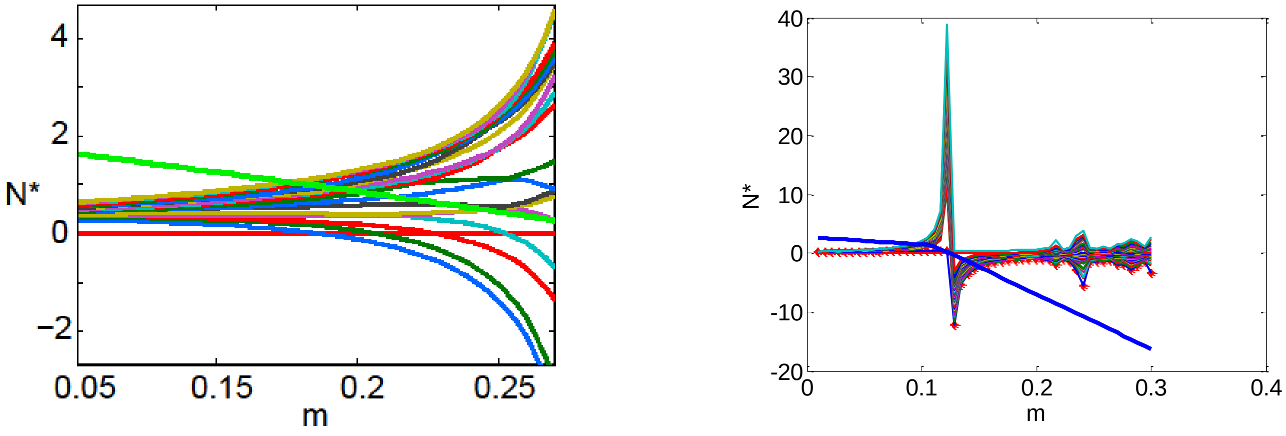

Supplementary Figure 5. (Reproduction of Supplementary Figure 19 from Stone (2016)).

LHS) Scenario I. Equilibrium abundances of CM-model for *n=20* species ($n_1 = n_2 = 10; c = 0.2,\ v = 0.4,\ q = 0.7$) plotted versus interaction strength *m*. Feasibility is lost at *m=0.18*. Critical eigenvalue of interaction matrix *A* (green line) zeroes at the "blow-up" point $m = 0.28$ where $|\boldsymbol{A}| = \boldsymbol{0}$ and stability is lost.

RHS) Scenario II $n_1 = n_2 = n/2$=30, c=0.1, v=0.7, q=1 Blow up occurs close to when $m \simeq c$. The minimum eigenvalue,here is real and plotted in blue as function of *m*. All population levels N_i^* plotted as a function of *m*.

First, note that not all equilibrium abundances decrease in the above figure (LHS). Second, note that the CM model has two different regimes which were investigated in great detail in Stone (2016).

Regime 1: When *c>>m*, the C-M model is in a regime where a huge amount of competition dominates the community dynamics. It would be unreasonable to expect mutualistic interactions to behave exactly as

predicted in the current model (Stone (2020)) where this interspecific competition is not allowed for at all. In particular, one will not always find the equilibria of all populations increasing as m increases when strong competition dominates the community and constrains the impact of mutualism, as seen in Supplementary Figure 5 LHS (i.e., Fig.S19a of Stone 2016).

Regime 2: Nevertheless, for situations when *c* does not exceed m excessively, the equilibrium populations never decrease with *m*. Instead the population equilibria of the CM model follow Supplementary Figure 5 RHS (Fig.S19 b above from Stone 2016), and *always increase with m*, until stability is lost. That is, in Regime 2 and competition is not excessive, the CM model behaves very similar to the model of this paper.

Finally, the most important advantage in working with the (non-CM) model in the present paper is that it aligns perfectly with some of the standard well-known random matrix models (Allesina, Coyte) where mutualism has been studied. Being perfectly compatible, it thus permits direct comparisons with related modelling studies in the literature.

Supplementary Notes 5: Feasibility calculations

Probability of feasibility: As in Ref.[2,3], for the n-species Lotka-Volterra system, let the interaction coefficients be of the form $a_{ij} = m + b_{ij}$ where $\mathrm{E}(b_{ij}) = 0$, and $Var\big(b_{ij}\big) = \sigma^2$. The equilibrium populations can be approximated by:

$$N_i^* \approx \kappa\big(1 + \sum_{j=1}^{n} b'_{ij}\big) = \kappa\,(1 + X_i),$$

where $b'_{ij} = b_{ij}/(1+m)$, $X_i = \sum_{j=1}^{n} b'_{ij}$ and the constant $\kappa>0$.

For $n \geq 5$ species, X_i is Normally distributed with: $E(X_i) = 0$ and $Var(X_i) = \frac{n\,\sigma^2}{(1+m)^2} = \gamma^2$.

The parameter γ proves to be important and represents the scaled disturbance level. Proceeding further, we can write the probability a single species has a positive equilibrium population is:

$$P(N_i^* > 0) = P(X_i > -1) = P(Z_i > -1/\gamma) = \Phi(1/\gamma) = \frac{1}{\sqrt{2}\,\pi} \int_{-\infty}^{1/\gamma} e^{-t^2}\,dt = p(\gamma)$$

where $Z_i \sim \mathrm{Normal}(0,1)$. For any level of disturbance γ, the probability of feasibility that all n-species have positive equilibrium populations is thus:

$$\mathrm{P(Feasible)} \cong \mathrm{p}(\gamma)^{\,n} = \Phi(1/\gamma)^{\,n} \quad . \tag{6}$$

Supplementary Figure 6 plots the theoretical probability of feasibility, and the estimate obtained from 500 simulations for each value of n, and γ.

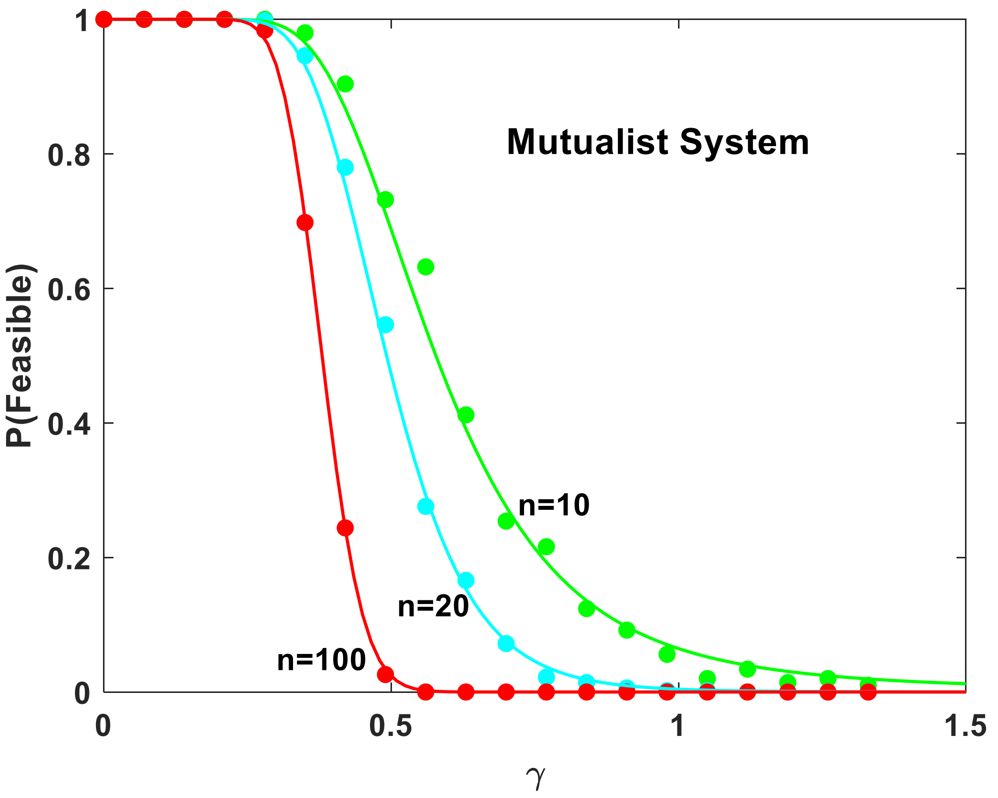

Supplementary Figure 6. The probability of feasibility, Pr(Feasible), as a function of disturbance γ, for *n*-species mutualism with different community sizes *n*=10, 20 and 100. Each probability marked by a circle is the proportion of feasible systems in 500 runs of equation (1). Analytical prediction according to the Methods are displayed as continuous curves. All feasible systems are stable. The mean interaction strength (m) for these simulations and number of species (*n)* present are: *n*=10, *m*=+0.05; *n*=20, *m*= +0.03*;* *n*=100; *m*=+0.009

Note that the mean interaction strength (m) for these simulations and number of species (n) present are: *n*=10, *m*=+0.05 (green); *n*=20, *m*= +0.03 (blue); *n*=100; *m*=+0.009 (red). These curves show the feasibility requirement for these mutualistic systems that $\gamma^2 = \frac{n\,\sigma^2}{(1+m)^2} \ll 1$, for large *n*. Thus feasible stable systems can be expected for limitation $m < 1/(n-1)$ [see Discussion, main text] and $\sigma^2 < 1/n$ approximately.

These feasibility predictions have much in common with those found for competitions systems[2]. For n <100, the feasibility constraint is not that dissimilar to May's stability condition for the stability of large random ecosystems, namely, $n\sigma^2 < 1$, But for larger systems, feasibility requires $n\sigma^2 \ll 1$ Note that for purely mutualistic systems having positive intrinsic growth rates, all feasible systems are stable and have a stable interaction matrix A[4]. The same appears to hold for systems discussed here which are not purely mutualistic, as pointed out by Roberts[5] and confirmed by numerical analyses. This generality needs to be explored further for the case that birth rates are not all positive.

Supplementary References

1 Stone, L. The Google matrix controls the stability of structured ecological and biological networks. *Nature communications* **7**, 12857 (2016).

2 Stone, L. The feasibility and stability of large complex biological networks: a random matrix approach. *Scientific reports* **8**, 8246 (2018).

3 Stone, L. Some problems of community ecology processes, patterns and species persistence in ecosystems. *https://figshare.com/articles/Some_problems_of_community_ecology_processes_patterns_and_species_persistence_in_ecosystems/4519409* (1988).

4 Su, W. & Guo, L. Interaction strength is key to persistence of complex mutualistic networks. *Complex Systems* **25**, 157-168 (2016).

5 Roberts, A. The stability of a feasible random ecosystem. *Nature* **251**, 607 (1974).
